# Distal Renal Tubular Acidosis as a Renal Manifestation of Sjögren’s Syndrome: A Case Report

**DOI:** 10.7759/cureus.109858

**Published:** 2026-05-29

**Authors:** Sangya Sharma, Aariez Khalid, Guillermo A Herrera, Emad Al Jaber

**Affiliations:** 1 Internal Medicine, University of South Alabama, Mobile, USA; 2 Internal Medicine, USA Health University Hospital, Mobile, USA; 3 Pathology, University of South Alabama, Mobile, USA; 4 Nephrology, University of South Alabama, Mobile, USA

**Keywords:** autoimmune nephropathy, recurrent hypokalemia, renal tubular acidosis (rta), renal tubular dysfunction, sjogren's syndrome

## Abstract

Distal renal tubular acidosis (dRTA) is a rare but recognized renal complication of Sjögren’s syndrome (SS), often resulting from autoimmune-mediated damage to acid-base transporters in the distal nephron. It commonly presents with electrolyte abnormalities including hypokalemia and metabolic acidosis. A 47-year-old woman with chronic kidney disease presented with recurrent hypokalemia, mild proteinuria, and sicca symptoms. Workup revealed non-anion gap metabolic acidosis, a positive urine anion gap, and renal potassium wasting consistent with dRTA. Autoimmune testing showed a high-titer ANA (1:1280) and elevated SSA (>8.0), confirming SS. Renal biopsy revealed mild interstitial fibrosis and glomerulosclerosis without evidence of tubulointerstitial nephritis or immune complex deposition. This finding supports prior evidence that SS-associated dRTA may occur secondary to functional tubular defects involving distal nephron transporters, even in the absence of overt inflammatory histologic changes. Acid-base disturbances improved with potassium and bicarbonate supplementation. This case highlights that significant tubular dysfunction in SS may occur despite minimal or absent histologic evidence of tubulointerstitial nephritis. dRTA in SS may result from autoimmune targeting of distal nephron transporters such as H⁺-ATPase and anion exchanger 1. Early recognition and supportive treatment are essential to prevent complications including nephrolithiasis, progressive kidney dysfunction, and life-threatening hypokalemia. SS should be considered in patients presenting with unexplained dRTA, and timely correction of electrolyte abnormalities is critical to optimizing outcomes.

## Introduction

Distal renal tubular acidosis (dRTA) in Sjögren’s syndrome (SS) is a well-documented but relatively uncommon extraglandular manifestation, and its underlying pathophysiology remains incompletely understood. Distal RTA (type 1 RTA) is characterized by impaired urinary acidification in the distal nephron, resulting in an inappropriately elevated urine pH (>5.5) despite systemic metabolic acidosis [1]. Clinically, this manifests as a non-anion gap hyperchloremic metabolic acidosis, frequently accompanied by hypokalemia, nephrocalcinosis, and nephrolithiasis due to hypocitraturia and impaired calcium handling [2]. In patients with SS, dRTA is commonly associated with tubulointerstitial nephritis (TIN), leading to dysfunction of distal tubular acidification mechanisms [3].

Proposed mechanisms of dRTA in SS include dysfunction of vacuolar-type H⁺-ATPases (V-ATPases) and chloride/bicarbonate exchangers such as anion exchanger 1 (AE1) located in alpha-intercalated cells of the distal convoluted tubules and collecting ducts. These transporters play a critical role in hydrogen ion secretion and maintenance of acid-base homeostasis [4]. Prior studies have demonstrated reduced or absent expression of V-ATPases and related transporters in patients with SS-associated dRTA, even in the absence of significant tubulointerstitial inflammation on renal biopsy [5]. Additionally, autoantibodies against V-ATPases have been reported in patients with SS-associated dRTA and may contribute to impaired tubular acidification [5]. Potassium homeostasis in the distal nephron is closely linked to acid-base regulation, and disturbances in distal tubular transport mechanisms may contribute to renal potassium wasting and hypokalemia in dRTA [6,7]. Clinically, dRTA in SS may present with recurrent hypokalemia, nephrolithiasis, nephrocalcinosis, and progressive renal impairment. We report a case of distal RTA in a patient with autoimmune serologic findings consistent with SS.

## Case presentation

A 47-year-old woman with a medical history significant for chronic kidney disease (CKD) stage III, chronic recurrent hypokalemia, sickle cell trait, dyslipidemia, and tobacco use presented to the University Hospital (UH) after a routine nephrology clinic visit revealed severe hypokalemia. Her serum potassium was found to be as low as 2.2 mmol/L (reference range: 3.5 to 5.2 mEq/L), and an electrocardiogram (ECG) demonstrated U waves. She was admitted for evaluation and management of nausea and vomiting in the setting of severe hypokalemia. 

Workup revealed distal (type 1) renal tubular acidosis (RTA), with a positive urine anion gap of 9, and an initial urine potassium-to-creatinine ratio (K/Cr) >13, suggestive of renal potassium wasting. Her potassium levels improved with supplementation during hospitalization and at subsequent clinic visits. 

Incidentally, she reported dry eyes and other sicca symptoms, raising suspicion for SS. Autoimmune workup revealed a positive ANA at a titer of 1:1280 and SSA > 8.0. Other markers, including SSB, anti-CCP, rheumatoid factor (RF), and complement levels (C3 and C4), were within normal limits or negative. The combination of non-anion gap metabolic acidosis, hypokalemia, and findings consistent with type 1 RTA was attributed to SS. The patient showed no evidence of systemic involvement of her SS at the time of evaluation. Renal workup revealed a progressive increase in urine protein-to-creatinine ratio, rising from 0.5 mg/g to 0.79 mg/g over six months, while the urine albumin-to-creatinine ratio remained <30 mg/g (Table 1). The kappa/lambda light chain ratio was within the renal reference range, and serum immunofixation electrophoresis showed no monoclonal proteins. One year prior, serum protein electrophoresis demonstrated a pattern consistent with chronic inflammation. 

**Table 1:** Urine Protein-to-Creatinine Ratio and Urine Albumin-to-Creatinine Ratio Over Six Months The urine protein-to-creatinine ratio and urine albumin-to-creatinine ratio were assessed over a six-month span, with levels stable between February and March, followed by a modest rise by July. Reference for UPCR: <0.15 mg/g normal, 0.15-0.50 mg/g moderately increased, >0.50 mg/g severely increased Reference for UACR: <30 mg/g normal, 30-300 mg/g moderately increased, >300 mg/g severely increased

Month & Year	Urine Protein-to-Creatinine Ratio (UPCR) in Milligrams/Gram (mg/g)	Urine Albumin-to-Creatinine Ratio (UACR) in Milligrams/Gram (mg/g)
February 2024	0.5	<0.30
March 2024	0.5	<0.30
July 2024	0.79	<0.30

**Figure 1:**
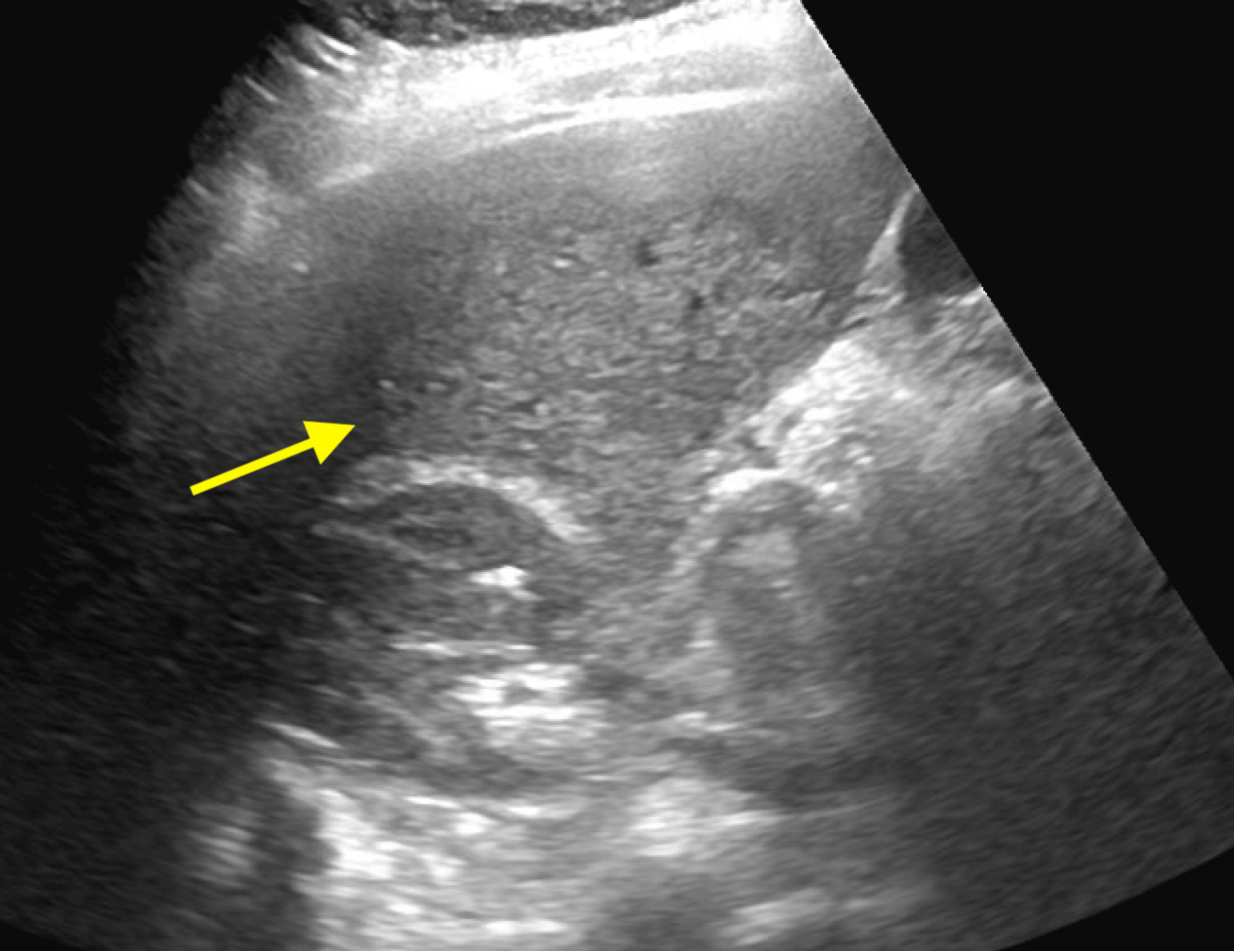
Renal Ultrasound of the Right Kidney Right kidney with mildly increased echogenicity and no hydronephrosis, stones, or nephrocalcinosis.

Renal ultrasound showed a left kidney with normal echogenicity and a right kidney with mildly increased echogenicity, without evidence of hydronephrosis, nephrolithiasis, or nephrocalcinosis (Figure 1).

**Figure 2:**
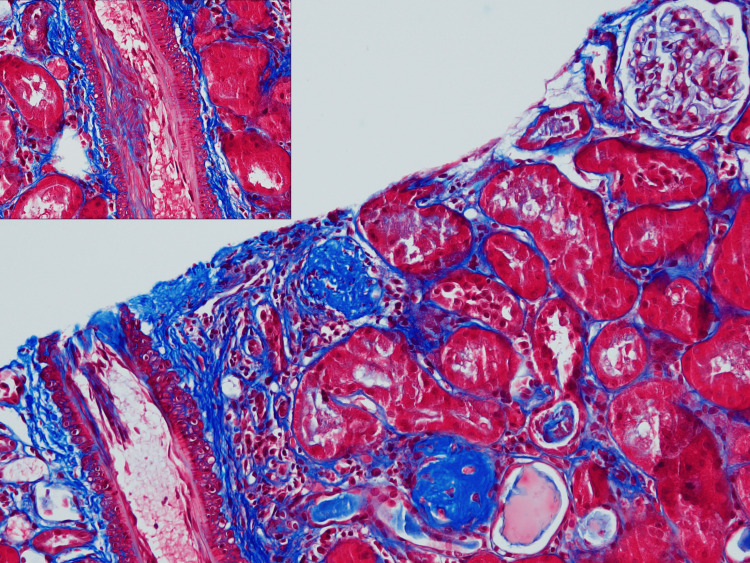
Trichrome-Stained Renal Biopsy, Shown at ×350 Magnification in the Main Image and ×750 Magnification in the Upper Left Inset Mild vascular sclerosis with 10-15% global glomerulosclerosis and focal interstitial fibrosis.

Given the presence of CKD, persistent proteinuria, and concern for possible autoimmune renal involvement, a renal biopsy was performed to evaluate for TIN or immune-mediated glomerular disease. Renal biopsy revealed mild vascular sclerosis, with 10-15% globally sclerosed glomeruli and mild, focal interstitial fibrosis. There was no evidence of TIN, and no immune complex deposition was identified (Figure 2).

Given the patient’s recurrent hypokalemia, non-anion gap metabolic acidosis, urinary potassium wasting, and positive autoimmune serologies including ANA and SSA, dRTA secondary to SS was considered the most likely diagnosis. Initial management included potassium chloride 40 mEq twice daily, sodium bicarbonate 1300 mg twice daily, and amiloride 5 mg daily, which was subsequently transitioned from potassium chloride and sodium bicarbonate to potassium citrate 30 mEq twice daily for ongoing alkali and potassium replacement. Amiloride was discontinued because of low-normal blood pressure. The patient was also counseled on maintaining a potassium-rich diet and referred to rheumatology for further evaluation and management of SS.

## Discussion

dRTA is a recognized extraglandular manifestation of SS, although the reported prevalence varies across studies. Previous reports suggest that up to 25% of patients with primary SS may demonstrate incomplete dRTA, characterized by impaired urinary acidification without overt systemic acidosis, while approximately 5% develop complete dRTA with clinically significant metabolic acidosis [8,9]. Renal involvement in SS may present with a wide range of manifestations including hypokalemia, muscle weakness, hypokalemic paralysis, nephrolithiasis, nephrocalcinosis, and progressive renal dysfunction [10]. Our patient presented with recurrent severe hypokalemia, non-anion gap metabolic acidosis, and renal potassium wasting, findings consistent with distal tubular dysfunction.

The pathophysiology of SS-associated dRTA is thought to involve autoimmune-mediated injury to alpha-intercalated cells within the distal nephron and collecting ducts. These cells are responsible for urinary acidification through the action of vacuolar-type H⁺-ATPases (V-ATPases) and chloride/bicarbonate exchangers such as AE1. Prior studies have demonstrated reduced or absent expression of these transporters in patients with SS-associated dRTA, suggesting that impairment of distal hydrogen ion secretion plays a central role in the development of metabolic acidosis [8]. In addition, autoantibodies directed against components of the acidification machinery have been identified in some patients, further supporting an autoimmune mechanism of tubular dysfunction.

Although TIN is commonly associated with renal involvement in SS, several reports have demonstrated that significant functional tubular impairment may occur even in the absence of substantial inflammatory changes on renal biopsy [8]. This was similarly observed in our patient, whose biopsy demonstrated mild interstitial fibrosis and glomerulosclerosis without evidence of active tubulointerstitial nephritis or immune complex deposition. The absence of marked inflammatory findings despite clinically significant dRTA highlights the possibility that molecular or functional transporter abnormalities may precede overt structural renal injury in some patients with SS.

The diagnostic evaluation of dRTA requires careful assessment of acid-base status and urinary electrolyte abnormalities. In our patient, the presence of non-anion gap metabolic acidosis, elevated urine pH, positive urine anion gap, and renal potassium wasting supported the diagnosis of distal RTA. Positive ANA and SSA serologies further supported the diagnosis of underlying SS. Similar presentations have been described in prior case reports in which severe hypokalemia and metabolic acidosis were among the earliest manifestations leading to recognition of SS [9].

Management primarily focuses on correction of hypokalemia and chronic metabolic acidosis through potassium and alkali supplementation. Potassium citrate supplementation may also help reduce complications related to hypocitraturia, including nephrolithiasis [2]. In patients with progressive renal involvement or significant systemic manifestations, corticosteroids or other immunosuppressive therapies may be considered to address the underlying autoimmune process [8]. Early recognition and treatment are important to prevent recurrent electrolyte disturbances, nephrolithiasis, and progressive renal dysfunction.

## Conclusions

dRTA is an important renal manifestation of SS and may present with recurrent hypokalemia and metabolic acidosis before significant structural renal abnormalities become apparent. Although this association has been previously described in the literature, this case reinforces the educational importance of recognizing characteristic laboratory findings, including non-anion gap metabolic acidosis, renal potassium wasting, elevated urine pH, and positive autoimmune serologies, as important diagnostic clues to underlying SS. Careful interpretation of these abnormalities may facilitate earlier diagnosis and treatment, helping reduce complications and preserve renal function.
